# Effects of *S*-Abscisic Acid on Reproductive Performance in Sows

**DOI:** 10.3390/ani16121885

**Published:** 2026-06-18

**Authors:** Daisuke Matsui, Maria Herrero, Iki Taketani, Tsuyoshi Tonoue, Koya Ueda, Ichiro Hagimori, Izuru Shinzato

**Affiliations:** 1 Sumitomo Biorational Company LLC, Libertyville, IL 60048, USA; 2 Sumitomo Chemical Co., Ltd., Tokyo 103-6020, Japan; 3 NAS Laboratory Co., Ltd., Chiba 286-0121, Japan

**Keywords:** *S*-abscisic acid, apocarotenoids, antioxidant, reproductive performance, sows

## Abstract

This study evaluated the effects of dietary *S*-abscisic acid (*S*-ABA) supplementation on reproductive performance and piglet growth in multiparous sows under commercial farming conditions. A total of 65 Landrace × Yorkshire multiparous sows were allocated to one of three dietary treatments: a basal diet (control); a basal diet supplemented with 1 ppm *S*-ABA; or a basal diet supplemented with 10 ppm *S*-ABA. The experimental period was set from weaning through the subsequent weaning (approximately 147 days), encompassing the non-pregnant, gestation, and lactation phases. Reproductive performance parameters, including the number of piglets born, the number of piglets weaned, and piglet body weight at birth and at weaning, were recorded, in addition to general health observations of sows and piglets. 1 ppm *S*-ABA supplementation significantly increased the number of piglets born compared to control (*p* < 0.05). Both 1 ppm and 10 ppm *S*-ABA supplementation resulted in a significantly higher number of piglets weaned (*p* < 0.05) and average piglet weaning weight (*p* < 0.01) compared with control, with no apparent differences in measured piglet survival outcomes among treatment groups. No additional benefits were observed when the supplementation level was increased from 1 ppm to 10 ppm. These findings suggest that dietary *S*-ABA supplementation at low inclusion levels may positively influence sow reproductive performance and piglet growth under practical feeding conditions.

## Simple Summary

*S*-abscisic acid (*S*-ABA) is a natural compound that is ubiquitously present in plants, and its role as a plant growth regulator has been well documented. Recent studies have suggested that *S*-ABA may improve antioxidant status and glucose metabolisms in animals; however, studies evaluating the effects of *S*-ABA on production performance in farm animals remain limited. In this article, we evaluated the effects of dietary *S*-ABA supplementation on the reproductive performance of sows. Sows were fed diets supplemented with *S*-ABA from weaning through the subsequent reproductive cycle, including gestation and lactation, under commercial farming conditions. Dietary supplementation with *S*-ABA suggested a potential improvement in sow reproductive performance.

## 1. Introduction

Apocarotenoids are organic compounds that occur widely in living organisms. They are generated through enzymatic or non-enzymatic cleavage of carotenoids, resulting in a diverse range of biologically active molecules. Among apocarotenoids, retinoids, including vitamin A and its derivatives, are the most extensively characterized [1].

*S*-abscisic acid (*S*-ABA), an apocarotenoid, is a natural plant growth regulator that has been identified in all plant species examined to date [2]. Abscisic acid has been shown to accumulate through de novo synthesis in ripening fruits such as tomatoes and avocado [3]. The presence of abscisic acid has also been reported in typical feed ingredients, such as barley [4], wheat [5], maize [6], and soybean [7], with concentrations ranging from 0.01 to 0.6 ppm in mature seeds. Considerably higher concentrations, up to 12.2 ppm, have been reported in developing soybean seeds [7]. Through de novo synthesis, *S*-ABA is recognized as a key regulator of plant growth and development, contributing to stress tolerance, fruit set, ripening, and senescence. As a ubiquitous bioactive component in plants, *S*-ABA plays a crucial role in plant responses to oxidative stress by enhancing antioxidant defense systems and mitigating oxidative damage [8].

In addition to its established functions in plants, recent studies have demonstrated antioxidative properties of *S*-ABA in animal models. Oral supplementation of *S*-ABA in rats and mice has been shown to reduce levels of reactive oxygen species (ROS) and malondialdehyde (MDA), while increasing antioxidant markers such as reduced glutathione (GSH) [9,10,11]. In weaned piglets, dietary supplementation with *S*-ABA (0.5–5.0 ppm) has been reported to improve antioxidant status as evidenced by a reduced erythrocyte GSSG:GSH ratio [12]. The safety of *S*-ABA has been evaluated primarily based on toxicological studies conducted in rodents and has been reviewed by regulatory authorities such as the European Food Safety Authority (EFSA) and the U.S. Environmental Protection Agency (EPA) [13,14]. These evaluations have recognized *S*-ABA as a compound of low toxicological concern. However, despite its established safety profile, reports evaluating the effects of *S*-ABA on production performance in livestock species remain limited.

In commercial swine production, reproductive efficiency of sows and growth performance of piglets are critical determinants of overall productivity [15]. In recent decades, genetic selection for increased prolificacy has markedly improved reproductive output in modern sows; however, this progress has also been accompanied by emerging challenges, including increased incidences of stillbirths, reduced piglet birth weight, and lower weaning weight of piglets [16,17,18]. These challenges are considered to be partly associated with increased metabolic demand and oxidative stress especially in highly prolific sows [19,20,21,22,23]. Accordingly, various nutritional strategies, particularly antioxidant-related materials such as vitamin E, selenium, plant-derived polyphenols, and carotenoids, have been investigated to support reproductive performance and piglet growth [24,25,26,27,28]. Nevertheless, additional bioactive nutritional compounds remain to be explored to support reproductive performance under modern commercial swine production systems.

Given that abscisic acid naturally occurs in plant-based feed ingredients commonly used in swine diets, understanding the effects of additional dietary *S*-ABA supplementation on sow performance is relevant from both nutritional and safety perspectives. Therefore, the objective of the present study was to evaluate the effects of dietary *S*-ABA supplementation on reproductive performance in multiparous sows under commercial farming conditions.

## 2. Materials and Methods

### 2.1. Animals and Experimental Design

The animal study protocol was approved by the Institutional Review Board of NAS Laboratories Co., Ltd. (protocol code 17-S033, approved on 19 April 2017). The study was conducted at a commercial pig farm in Japan using 65 multiparous LW (Landrace × Large White) sows (parity 1–8; age 1.1–4.4 years). Prior reproductive performance was evaluated, and sows were allocated to one of three dietary treatment groups to minimize differences among groups in average age, average parity, and average numbers of piglets born and weaned in the previous farrowing, as shown in Table 1 and Table 2. Sows received either a basal diet (control), the basal diet supplemented with 1 ppm *S*-ABA, or the basal diet supplemented with 10 ppm *S*-ABA. Sows were fed the experimental diets from weaning until the subsequent weaning, covering a total experimental period of approximately 147 days, including the non-pregnant period (5 days), gestation (approximately 114 days), and lactation (28 days).

### 2.2. Feeding and Management

A commercial breeding sow diet formulated according to standard farm feeding practices and primarily based on corn and soybean meal was prepared as a basal diet. Feed was offered at a fixed allowance, and the allowance was adjusted according to the reproductive stage following routine farm management and was applied equally across all dietary treatment groups. During the post-weaning non-pregnant period, daily feed allowance ranged from 0.7 to 3.0 kg per sow. During gestation, sows were restrictively fed 2.5 to 3.0 kg per sow per day. From farrowing through the lactation period, daily feed allowance ranged from 0.7 to 6.0 kg per sow, depending on the lactation stage.

*S*-abscisic acid (*S*-ABA; purity ≥ 95%) was provided by Valent BioSciences LLC (Libertyville, IL, USA) and incorporated into the basal diet by thoroughly mixing using a ribbon mixer. Water was provided ad libitum throughout the experimental period.

For suckling piglets, no solid feed was provided until 14 days after birth. From 15 days of age, a commercial creep feed mainly based on dried whey, skim milk powder, and wheat flour was offered at approximately 100 g per sow per day and was gradually increased based on piglet feed intake, up to a maximum of 500 g per day until weaning. Creep feed was provided in piglet-only feeders designed to prevent access by sows, and feeding management and feeder design were identical across all dietary treatment groups. The composition of the sow diet and the creep feed is shown in Table 3 and Table 4, respectively.

### 2.3. Data Collection

Reproductive performance and piglet growth data were collected throughout the experimental period. Pregnancy and farrowing outcomes were recorded for all sows enrolled in the study. For sows that farrowed, the total number of piglets born (including stillborn piglets), the number of piglets at the start of suckling, and the number of piglets weaned were recorded for each sow. Piglet body weight was measured individually at birth and at weaning (28 days of age), and mean values were calculated on a per-sow basis. General health conditions of sows and suckling piglets were monitored throughout the study period according to routine farm practices.

### 2.4. Statistical Analysis

For reproductive performance measures, individual sows were considered the experimental unit. Data for litter size and piglet body weight were analyzed using one-way analysis of variance (ANOVA). When significant differences were detected, comparisons between the control and *S*-ABA-supplemented groups were conducted using Dunnett’s test. Pregnancy rate was analyzed using Fisher’s exact test. Statistical significance was declared at *p* < 0.05 (*) and *p* < 0.01 (**). All statistical analyses were performed using BellCurve for Excel (Version 2.14; Social Survey Research Information Co., Ltd., Tokyo, Japan).

During animal allocation, efforts were made to balance treatment groups with respect to average parity and previous reproductive performance (Table 1 and Table 2). Parity was therefore not included as a covariate in the statistical model, as group characteristics were prospectively balanced and the study was designed as an exploratory trial under commercial farming conditions. However, potential residual effects of parity cannot be fully excluded and should be considered when interpreting the results.

## 3. Results and Discussion

A total of 65 sows were enrolled in the study, including 21 sows in the control group, 22 sows in the *S*-ABA 1 ppm group, and 22 sows in the *S*-ABA 10 ppm group. Among these, 17, 20, and 20 sows, respectively, became pregnant following artificial insemination and subsequently farrowed. This corresponded to pregnancy rates of 81.0%, 90.9%, and 90.9% for the control, 1 ppm, and 10 ppm groups, respectively, with no statistically significant differences among groups (*p* > 0.05, Fisher’s exact test). The remaining 4, 2, and 2 sows, which did not become pregnant and returned to estrus, were excluded from the study. All reproductive performance and piglet growth data presented below were therefore based on these farrowed sows. One sow in the *S*-ABA 10 ppm group experienced premature farrowing at 110 days of gestation; however, all other sows farrowed within the normal gestation range of 113 to 117 days. No other abnormalities were observed based on general health observations during the experimental period, suggesting that dietary *S*-ABA supplementation was well tolerated under commercial farming conditions.

As shown in Table 5, sows receiving diets supplemented with *S*-ABA produced a greater number of piglets at farrowing than those in the control group. This finding suggests that dietary *S*-ABA supplementation may positively influence reproductive output under the conditions of this study. This increase was statistically significant in the *S*-ABA 1 ppm group (*p* < 0.05), while a similar numerical trend was observed in the *S*-ABA 10 ppm group (*p* = 0.067). The absence of a clear dose-dependent response between 1 ppm and 10 ppm suggests that the biological response to dietary *S*-ABA may reach a plateau at low inclusion levels, or that the effects of *S*-ABA on reproductive outcomes are not strictly dose-responsive within the tested range. However, additional studies using a broader range of supplementation levels are needed to confirm the definitive dose–response relationship. Importantly, the number of stillborn piglets and piglets culled before the start of suckling did not differ among treatments (*p* > 0.05), indicating that the increased litter size observed in *S*-ABA-supplemented sows was not accompanied by elevated perinatal losses.

Consistent with these findings, the number of piglets at the start of suckling and the number of piglets weaned per sow were significantly higher in both *S*-ABA-supplemented groups compared with the control group (*p* < 0.05). Piglet survival during the suckling period was generally high across all treatments, and only a small number of piglets were removed with no apparent treatment-related differences. Pre-weaning mortality due to crushing was limited and comparable among treatments (2, 3, and 2 piglets in the control, 1 ppm, and 10 ppm *S*-ABA groups, respectively), and a small number of piglets were removed due to poor growth (1, 3, and 2 piglets, respectively). These observations suggest that dietary *S*-ABA supplementation may support improved reproductive efficiency, with no apparent differences in measured piglet survival outcomes among treatment groups.

The average piglet body weight at birth and at the start of suckling did not differ among dietary treatments (*p* > 0.1) as shown in Table 6. In contrast, the average piglet body weight at weaning was significantly greater in both *S*-ABA 1 ppm and *S*-ABA 10 ppm groups compared with the control group (*p* < 0.01), suggesting a potential improvement in early growth performance. As observed for reproductive parameters, increasing the *S*-ABA supplementation level from 1 ppm to 10 ppm did not result in additional improvements in piglet growth under the conditions tested, suggesting limited additional benefit at the higher inclusion level. Because milk yield, colostrum production, or related maternal physiological parameters were not measured, the present study cannot distinguish whether the greater weaning weight was primarily associated with prenatal effects, lactational effects, or both.

The mechanisms underlying the observed improvements in sow reproductive performance and piglet growth cannot be fully elucidated based on the present study. Reproductive sows are known to experience substantial physiological challenges during gestation and lactation, including increased oxidative stress and alterations in glucose and energy metabolism particularly in metabolically active tissues such as the uterus, placenta, and mammary glands [19,20,29]. A recent review [30] summarized that maternal oxidative stress can impair placental function and negatively affect fetal survival and development, thereby contributing to poorer reproductive outcomes such as reduced litter size and litter weight. Considerable evidence in mammals indicates that oxidative stress is closely associated with reduced insulin sensitivity and impaired glucose metabolism [31], which may have important implications for energy partitioning during physiologically demanding states such as gestation and lactation. In sows, reduced maternal glucose tolerance during late gestation has been associated with increased postnatal pig mortality [32]. In line with this context, a study in sows has suggested that dietary supplementation with plant-derived polyphenols can concurrently improve oxidative status and alleviate insulin resistance, particularly during late gestation [33].

Previous studies have suggested that *S*-ABA may enhance antioxidant status in rodents [9,10,11] and pigs [12], which could contribute to improved reproductive outcomes by mitigating oxidative stress during gestation and lactation. In addition, emerging evidence indicates that *S*-ABA may improve insulin sensitivity and glucose utilization [34,35,36,37], raising the possibility that improved energy metabolism could play a role in supporting fetal development and milk production. However, these mechanisms remain speculative and warrant further investigation.

Improvements in litter size and average piglet weaning weight were observed, with no apparent differences in piglet survival or other measured outcomes among treatment groups. Collectively, these findings suggest that dietary *S*-ABA supplementation may contribute to improved reproductive performance under practical production conditions. However, this study should be regarded as an exploratory evaluation conducted under commercial farming conditions. Additional studies are warranted to confirm the reproducibility of the parameters assessed in this study, as well as to evaluate other aspects of reproductive performance such as weaning-to-estrus interval and subsequent reproductive outcomes, and to clarify the underlying biological mechanisms, including whether the observed effects are associated with reduced maternal oxidative stress and improved glucose metabolism, which were not directly assessed in the present study.

## 4. Conclusions

Under the conditions of this study, dietary supplementation with *S*-abscisic acid (*S*-ABA) resulted in improvements in sow reproductive performance and piglet growth, including increased litter size and higher weaning weights. Supplementation with 1 or 10 ppm *S*-ABA did not result in any adverse effects on the measured parameters in sows or piglets evaluated in this study. No additional benefits were observed when the supplementation level was increased from 1 ppm to 10 ppm, suggesting that low dietary inclusion levels may be sufficient to elicit physiological responses relevant to reproductive performance. These results suggest that *S*-ABA was well tolerated under the commercial production conditions of this study and may offer potential benefits for reproductive performance. However, as this study was conducted as an exploratory trial under commercial farming conditions, further studies are warranted to confirm these findings and to elucidate the underlying biological mechanisms.

## Figures and Tables

**Table 1:** Baseline characteristics of sows assigned to each treatment group, including age, parity, and previous reproductive performance.

Treatment	Number of Sows Allotted	Average Age (Years)	Average Parity	Average Number of Piglets in Previous Farrowings	
				Born	Weaned
Control	21	2.4 ± 0.9	3.3 ± 1.9	10.7 ± 1.2	9.8 ± 1.0
*S*-ABA 1 ppm	22	2.3 ± 0.9	3.2 ± 1.8	10.8 ± 1.0	9.6 ± 0.9
*S*-ABA 10 ppm	22	2.3 ± 0.8	3.1 ± 1.5	10.7 ± 1.3	9.7 ± 1.1

Values are expressed as mean ± SD.

**Table 2:** Distribution of sows by parity in each treatment group.

Treatment	Parity 1	Parity 2	Parity 3	Parity 4	Parity 5	Parity 6	Parity 7	Parity 8
Control	4	5	3	3	4	1	0	1
*S*-ABA 1 ppm	3	8	3	2	3	2	1	0
*S*-ABA 10 ppm	2	7	5	5	0	3	0	0

**Table 3:** Composition of the commercial sow diet, including ingredient composition and nutrient content.

Ingredients	
Grains (corn)	59%
Oilseed meal (soybean meal, rapeseed meal)	16%
Bran & by-products (rice bran, DDGS)	8%
Animal protein (fishmeal)	2%
Others (bakery by-products, alfalfa meal, calcium carbonate, animal fat, calcium phosphate, sodium chloride, feed-grade yeast, feed additives ^1^)	15%
Nutrient Composition	
Crude Protein	≥15.0%
Crude Fat	≥2.0%
Crude Fiber	≤10.0%
Crude Ash	≤10.0%
Calcium	≥0.80%
Phosphorus	≥0.50%
Total Digestible Nutrients	≥74.0%

^1^: Feed additives included iron peptides, ferrous sulfate, copper peptides, copper sulfate, magnesium oxide, manganese carbonate, zinc peptides, zinc carbonate, cobalt carbonate, calcium iodate, vitamin A, vitamin D3, vitamin E, vitamin B1, vitamin B2, folic acid, biotin, vitamin K3, 25-hydroxycholecalciferol, taurine, ethoxyquin, choline, phytase, and lysine.

**Table 4:** Composition of the commercial creep feed used in the study, including ingredient composition and nutrient content.

Ingredients	
Animal-derived ingredients (dried whey, skim milk powder, animal plasma protein, fishmeal, whey protein concentrate)	47%
Grains (wheat flour, bread crumbs, wheat, heat-treated corn)	37%
Oilseed meal (extruded dehulled soybean meal, soy protein concentrate)	3%
Others (animal fat, sucrose, glucose, lactose, calcium phosphate, calcium carbonate, citric acid, sodium chloride, medium-chain fatty acids from coconut oil, monk fruit extract, diatomaceous earth, Torula yeast, silica, cranberry extract, hydrogenated oil, sodium butyrate, lactic acid, koji fermentation product, cellulose, galacto-oligosaccharide syrup, anise seed oil, anise seeds, milk thistle, garlic, zeolite, and grape seed extract, feed additives ^1^)	13%
Nutrient Composition	
Crude Protein	≥21.5%
Crude Fat	≥4.0%
Crude Fiber	≤2.0%
Crude Ash	≤8.5%
Calcium	≥0.70%
Phosphorus	≥0.60%
Total Digestible Nutrients	≥87.0%

^1^: Feed additives included vitamin A, vitamin D3, vitamin E, vitamin K3, vitamin B1, vitamin B2, vitamin B6, vitamin B12, vitamin C, niacin, pantothenic acid, choline, folic acid, biotin, manganese peptides, zinc peptides, iron peptides, copper peptides, manganese carbonate, zinc carbonate, sodium sulfate, ferrous sulfate, copper sulfate, cobalt sulfate, magnesium sulfate, calcium iodate, methionine, lysine, threonine, tryptophan, L-valine, monosodium glutamate, taurine, protease, fiber-degrading enzymes, β-glucanase, phytase, Bacillus cereus, Clostridium butyricum, flavoring agents, binders, and ethoxyquin.

**Table 5:** Effects of dietary *S*-ABA supplementation on reproductive performance of sows, including litter size and piglet survival.

Treatment	Piglets Born	Stillborn Piglets	Piglets Culled Before Suckling ^1^	Piglets at the Start of Suckling	Piglets Weaned	Weaning Rate (%)
Control	12.1 ± 1.8	0.7 ± 0.9	0.4 ± 0.5	11.1 ± 1.0	10.9 ± 1.0	98.5 ± 3.4
*S*-ABA 1 ppm	14.0 ± 2.2 *	1.0 ± 1.0	0.7 ± 0.9	12.3 ± 1.5 *	12.0 ± 1.5 *	97.7 ± 4.3
*S*-ABA 10 ppm	13.6 ± 2.3	0.6 ± 0.9	0.6 ± 0.6	12.4 ± 1.6 *	12.2 ± 1.6 *	98.1 ± 3.4

Values are expressed as mean ± SD. * Significantly different from the control group (*p* < 0.05; Dunnett’s test). ^1^: Piglets culled before suckling include piglets removed from the study prior to the onset of suckling due to poor growth or viability.

**Table 6:** Effects of dietary *S*-ABA supplementation on piglet body weight (kg) at birth, at the start of suckling, and at weaning.

Treatment	At Birth	At the Start of Suckling	At Weaning
Control	1.40 ± 0.26	1.45 ± 0.23	8.00 ± 0.93
*S*-ABA 1 ppm	1.31 ± 0.21	1.38 ± 0.18	9.01 ± 0.50 **
*S*-ABA 10 ppm	1.33 ± 0.20	1.38 ± 0.18	9.16 ± 0.86 **

Values are expressed as mean ± SD. ** Significantly different from the control group (*p* < 0.01; Dunnett’s test).

## Data Availability

All data generated during this study are available upon reasonable request from the corresponding author.
